# Calreticulin: a potential diagnostic and therapeutic biomarker in gallbladder cancer

**DOI:** 10.18632/aging.202488

**Published:** 2021-02-11

**Authors:** Jianwen Ye, Lei Qi, Zhicheng Du, Long Yu, Kunlun Chen, Renfeng Li, Ruo Feng, Wenlong Zhai

**Affiliations:** 1 Department of Hepatobiliary and Pancreatic Surgery, The First Affiliated Hospital of Zhengzhou University, Zhengzhou 450052, Henan Province, China; 2 Department of Pharmacy, The First Affiliated Hospital of Zhengzhou University, Zhengzhou 450052, Henan Province, China; 3 Department of Histology and Embryology, Medical College of Zhengzhou University, Zhengzhou 450052, Henan Province, China; 4 Key Laboratory of Digestive Organ Transplantation of Henan Province, Open and Key Laboratory of Hepatobiliary and Pancreatic Surgery and Digestive Organ Transplantation at Henan Universities, Zhengzhou Key Laboratory of Hepatobiliary and Pancreatic Disease and Organ Transplantation, Zhengzhou 450052, Henan Province, China

**Keywords:** gallbladder cancer, calreticulin, biomarker, PI3K/Akt pathway

## Abstract

Recent studies suggested that calreticulin (CRT) has an important role in the progression of various types of cancer. Our previous study suggested that CRT was upregulated and acted as an oncogene in hepatocellular carcinoma. However, the role of CRT in gallbladder cancer (GBC) remains unclear. The expression level of CRT was upregulated in GBC tissues in comparison with adjacent non-tumor tissues and chronic cholecystitis tissues. Moreover, CRT expression was found to be correlated with the tumor size. Knockdown of CRT inhibited cell proliferation, induced apoptosis, arrested cell cycle and resulted in decreased resistance to gemcitabine, which was mediated by the inactivation of the PI3K/Akt pathway. Collectively, the present results suggested a potential role of CRT in GBC progression and provided novel insights into the mechanism underlying the CRT-mediated chemosensitivity in GBC cells.

## INTRODUCTION

Gallbladder cancer (GBC) is the fifth most common gastrointestinal cancer and the most aggressive malignant tumor of the bile tract cancer with a 5-year survival rate of 5% worldwide [1, 2]. Previous studies showed that GBC is difficult to diagnose at early stage due to the lack of specific symptoms, and GBC is characterized by a rapid progression. Complete surgical resection (R0) is the only effective way to treat patients with GBC. Although new examination and surgical techniques have been developed to diagnose and treat patients with GBC, only <30% of patients with GBC are considered to be surgical candidates [3, 4]. Moreover, due to its aggressive biological features and high rate of recurrence and metastasis, the prognosis of those patients remains poor [5]. Therefore, chemotherapy is indispensable for patients with unresectable GBC. Gemcitabine (GEM) is currently the standard first-line anticancer agent to treat many types of cancer, including GBC [6]. However, accumulating evidence from clinical trials showed that the response rate in patients with GEM is not satisfactory [7, 8]. Recent studies showed that targeting genes involved in drug-resistance may improve the therapeutic effects of GEM in various types of cancer, including pancreatic cancer [8] and bile tract cancer [9]. Therefore, the identification of new resistance-associated genes is necessary to increase the response rate.

Accumulating evidence demonstrated that aberrant gene expression in endoplasmic reticulum (ER), such as GRP78, a chaperone gene in ER, leads to chemoresistance in various cancer types [10, 11]. Calreticulin (CRT) is a multi-functional molecular chaperone mainly residing in the ER and is involved in the regulation of multiple cellular biological process, including Ca^2+^ homeostasis, immune response, cell adhesion, migration and malignant formation [12, 13]. Previous studies demonstrated that the expression of CRT is upregulated in many types of cancer, such as lung [14], pancreatic [15] and esophageal cancer [13]. Our previous study suggested that CRT is upregulated in hepatocellular carcinoma and a higher expression level of CRT is associated with tumor metastasis [16, 17]. Notably, CRT is frequently mutated in myeloproliferative neoplasms and targeting this gene may improve the treatment of myeloproliferative neoplasms [18, 19]. In addition, Sheng et al [15] showed that inhibition of CRT decreased the chemoresistance of pancreatic cancer, and previous studies demonstrated that CRT regulates the ERK/MAPK [20], NF-κB [21] and STAT3 [22] pathways. These previous studies indicated that CRT may play a key role in tumor progression in various types of cancer. However, the role of CRT in GBC remains unclear. In the present study, the expression levels of CRT in GBC tissues were found to be higher compared with paired non-tumor tissues and chronic cholecystitis tissues. In addition, a correlation was identified between the expression level of CRT and the clinicopathological features of patients with GBC. Moreover, CRT was found to act as an oncogene in GBC, and inhibition of CRT enhanced the chemosensitivity of GBC to GEM by inactivating the PI3K/Akt pathway.

## RESULTS

### CRT expression is upregulated in GBC tissues and associated with poor prognosis

**Figure 1:**
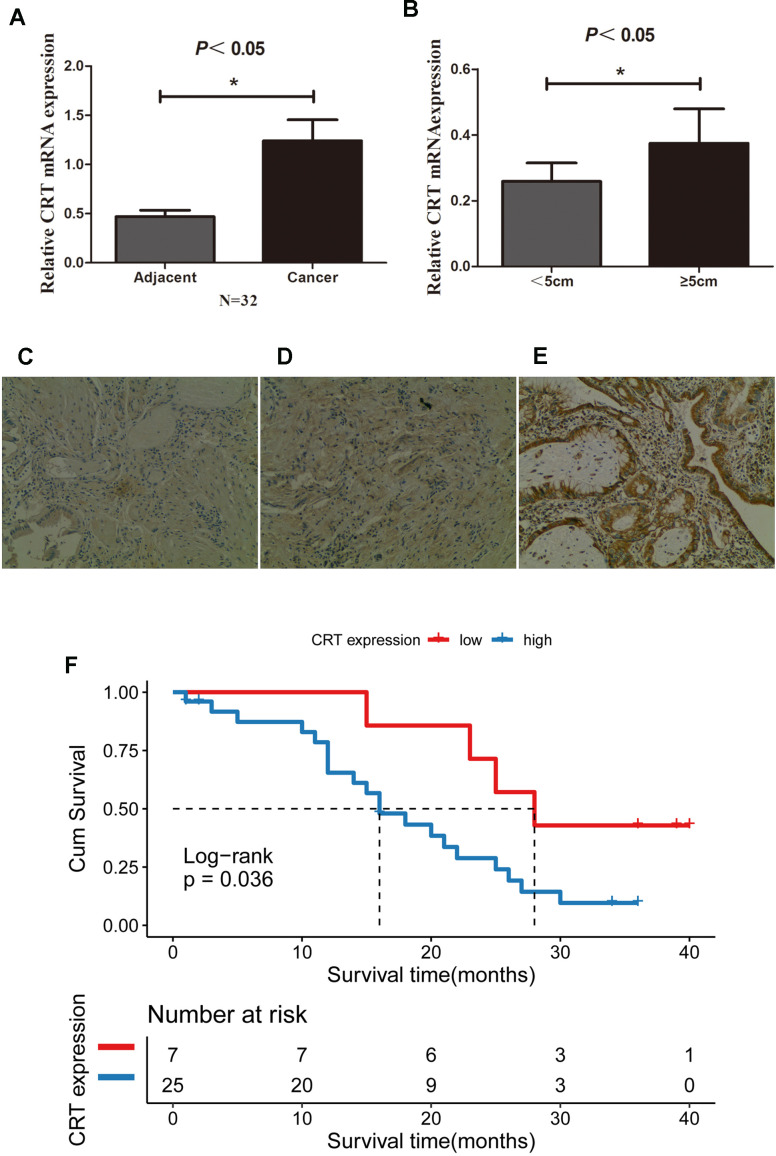
**CRT is upregulated in GBC tissues and is associated with poor prognosis.** (**A**) Relative mRNA expression of CRT in 32 paired adjacent and cancer tissues from patients with GBC. (**B**) Relative expression of CRT in tumor with a size <5cm and ≥5cm in patients with GBC. (**C**) IHC staining results for CRT in cholecystitis samples. (**D**) IHC staining results for CRT in adjacent peri-cancerous tissues. (**E**) IHC staining results for CRT in GBC cancer tissues (magnification x100). (**F**) Kaplan-Meier analyses of overall survival in 32 GBC patients based on CRT expression level of GBC tissues.^*^P<0.05 vs. adjacent tissue or vs. tumor with a size<5cm. CRT, calreticulin; GBC, gallbladder cancer.

The CRT mRNA and protein level in 32 paired GBC and adjacent normal gallbladder tissues were examined using RT-qPCR and IHC. The present results suggested that the expression of CRT was significantly higher in 25/32 GBC tissues compared with adjacent normal gallbladder tissues and chronic cholecystitis tissues (Figure 1A, B). In addition, the mRNA expression of CRT was higher in patients with a tumor size≥5 cm compared with patients with a tumor size <5cm. In addition, the expression of CRT in gallbladder tissues was also detected by IHC (Figure 1C–1E). The expression of CRT was increased in GBC tissues, which was consistent with the results obtained from the RT-qPCR analysis. Besides, patients with high CRT expression (median survival time = 16months, had an obviously poorer overall survival rate than those with low CRT expression (median survival time =28 months, P<0.05 log-rank test:χ^2^ =4.414) (Figure 1F).

### Relationship between CRT expression and the clinicopathological data of patients with GBC

The associated between CRT and clinicopathological data was also analyzed. Notably, as shown in Table 1, aberrant expression of CRT was found to be associated with tumor size. However, the expression of CRT was not associated with lymph node metastasis, TNM stage, histological grade or other clinicopathological parameters. Besides, Cox analysis indicated that CRT expression was independent predictor of poor prognosis in GBC patients (P<0.05). Compared with patients with low CRT expression, patients with high CRT expression of GBC have a higher risk of death (Table 2). The present results suggested that CRT play an important role in the progression of GBC.

**Table 1:** Relationship between CRT expression and clinicopathological features in GBC cases.

**Variables**	**Case number**	**CRT expression**		**P-value**
		**Low**	**High**	
Age(years)				
<60	9	2	7	0.657
≥60	23	5	18	
Gender				
Female	17	3	14	0.424
Male	15	4	11	
Histological grade				
Well and moderately	22	5	17	0.624
Poorly	10	2	8	
N status				
N0	19	5	14	0.389
N1/2	13	2	11	
Stone				
No	28	6	22	0.648
Yes	4	1	3	
Tumor size(cm)				
<5	21	7	14	0.035
≥5	11	0	11	
Clinical stage				
I-II	7	2	5	0.489
III-IV	25	5	20	

**Table 2:** Univariate and multivariate analysis for over survival.

**Variable**		**Univariable analysis**				**Multivariable analysis**			
		**95%CI for Exp(B)**				**95%CI for Exp(B)**			
		HR	Lower	Upper	P value	HR	Lower	Upper	P value
CRT expression	High vs Low	3.0	1.011	8.905	0.048	7.795	1.916	31.709	0.004
Age(year)	≤60 *vs* >60	0.525	0.224	1.228	0.137	0.227	0.072	0.718	0.012
Gender	Female *vs* Male	0.793	0.355	1.774	0.573	1.570	0.585	4.211	0.370
Histological grade	Well and moderately vs Poorly	0.870	0.372	2.037	0.749	0.377	0.126	1.132	0.082
N status	N0vsN1/2	1.405	0.613	3.221	0.422	1.380	0.488	3.906	0.544
Stone	No vs Yes	2.039	0.679	6.126	0.204	5.502	1.328	22.798	0.019
Tumor size(cm)	<5 vs ≥5cm	1.190	0.518	2.730	0.682	0.383	0.113	1.299	0.124
TNM	I-II vs III-IV	1.574	0.580	4.275	0.374	2.676	0.722	9.916	0.141

### Knockdown of CRT inhibits the proliferation of GBC-SD and NOZ cells

**Figure 2:**
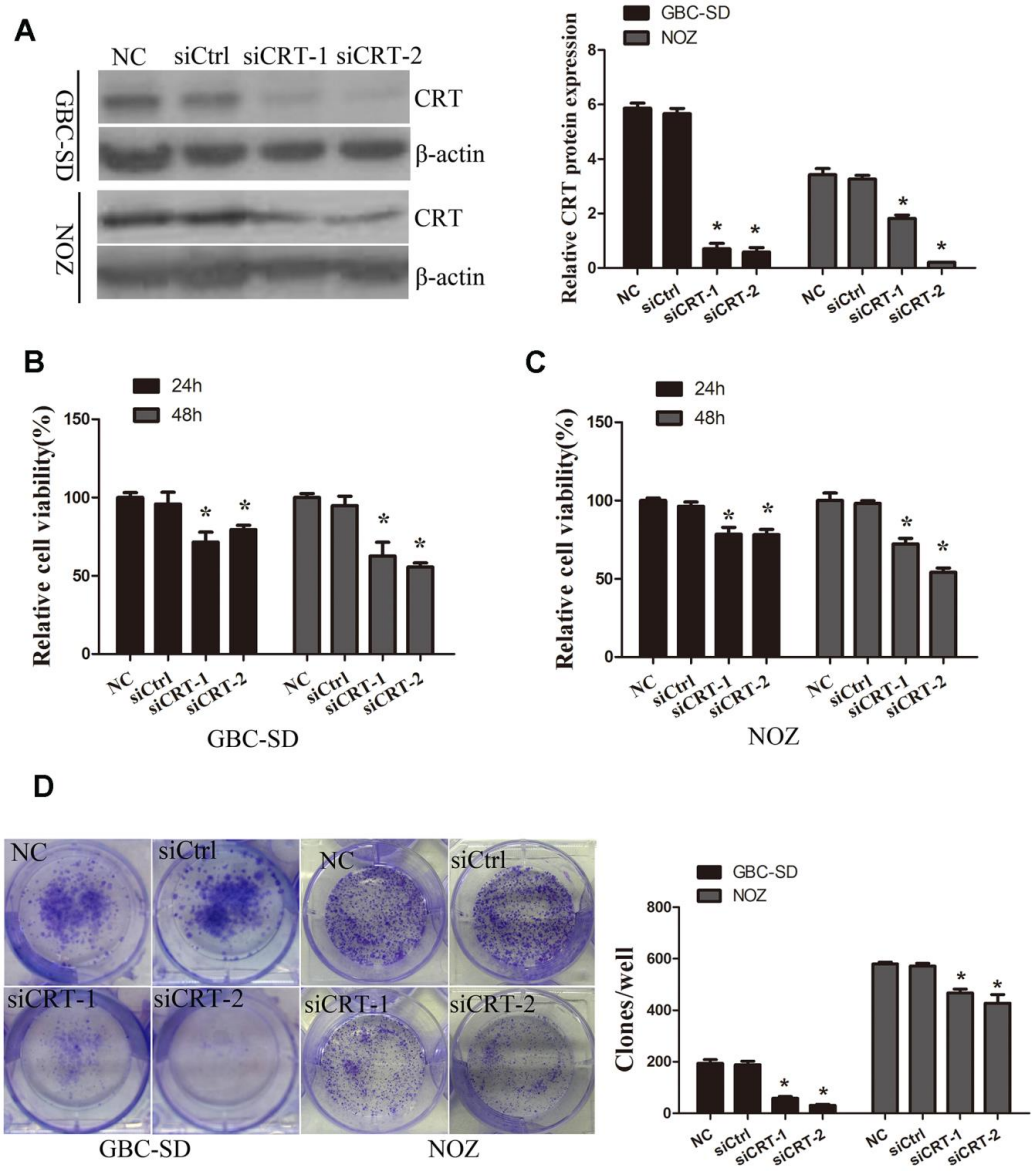
**Knockdown of CRT significantly inhibits cell proliferation *in vitro*.** (**A**) CRT expression following knockdown was confirmed by western blot assay. (**B**, **C**) CCK-8 assay was performed to analyze the proliferation of GBC-SD and NOZ cells. (**D**) Cell colony formation ability was detected by colony formation assay.^*^P<0.05 vs. negative control. CRT, calreticulin; GBC, gallbladder cancer.

In the present study, the role of CRT in the cell biological features of gallbladder cancer cells GBC-SD and NOZ was investigated. To examine the role of CRT in GBC-SD and NOZ cells, siCRT was used for downregulating the expression of CRT. As shown in Figure 2A, western blot analysis was performed to confirm the knockdown efficiency. Next, the effect of CRT knockdown on cell proliferation and colony formation was investigated. As shown in Figure 2B–2D, knockdown of CRT in GBC-SD and NOZ cells significantly inhibited cell proliferation and decreased the number and size of the colonies.

### Knockdown of CRT induces cell apoptosis and cell cycle arrest in GBC-SD and NOZ cells

**Figure 3:**
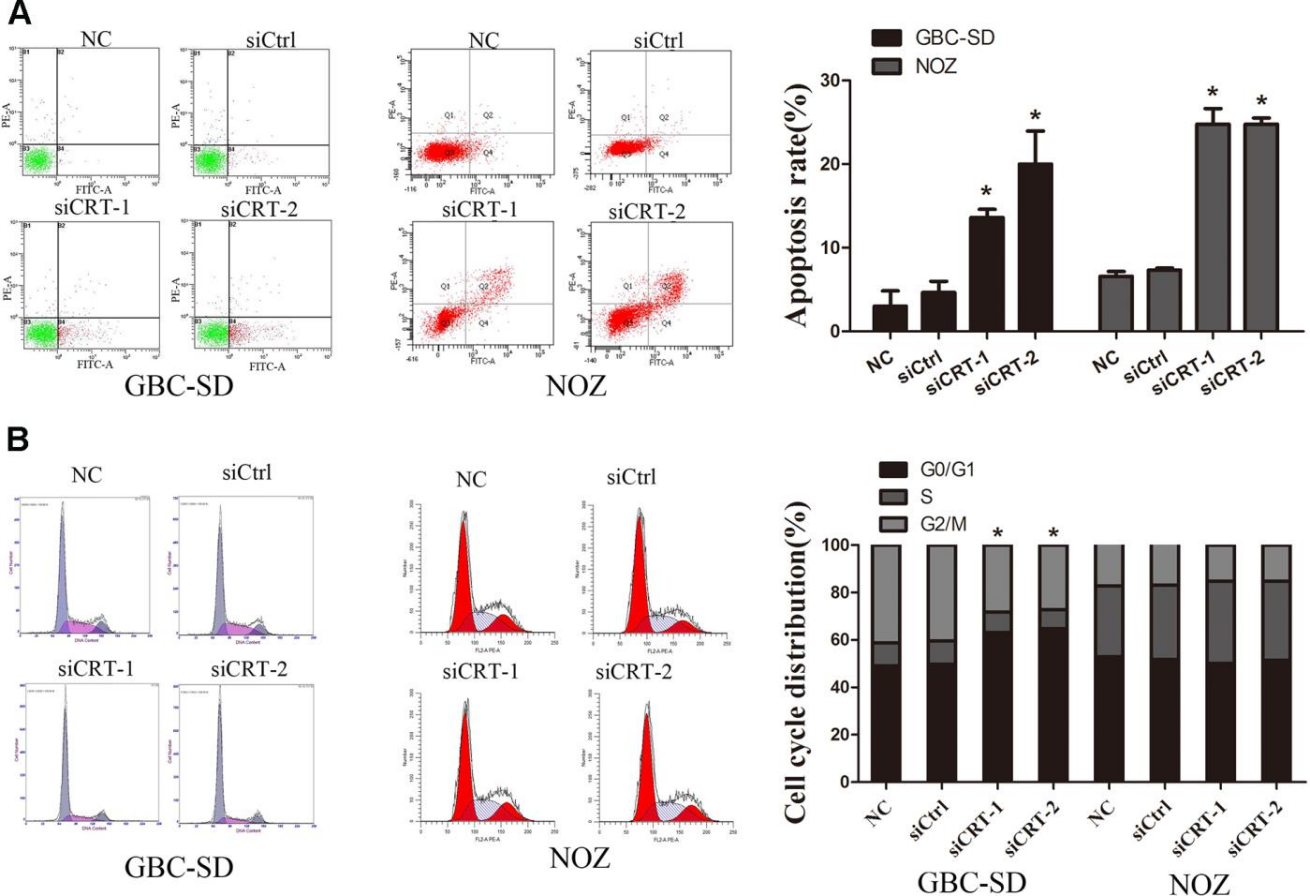
**Knockdown of CRT significantly induced cell apoptosis and cell cycle arrest.** (**A**) Cell apoptosis was detected by flow cytometry. (**B**) Cell cycle progression. ^*^P<0.05 vs. negative control. CRT, calreticulin; GBC, gallbladder cancer.

In the present study, the mechanisms underlying the CRT-mediated inhibition of proliferation were investigated. The apoptotic rate of cells transfected with siCRT was analyzed by flow cytometry. The apoptotic rates in GBC-SD cells in the siCRT-1 and siCRT-2 groups were 13.6±1.0% and 20.0±4.0%, respectively, whereas the rates in the negative control (NC) and siCtrl groups were 3.0±1.8% and 4.7±1.3%, respectively. Similarly, the apoptotic rates in NOZ cells in the siCRT-1 and siCRT-2 groups were 24.8±1.8% and 24.8±0.7%, respectively, whereas the rates in the negative control (NC) and siCtrl groups were 6.6±0.6% and 7.4±0.2%, respectively. The present results suggested that transfection with siCRT induced cell apoptosis (Figure 3A). In addition, the effect of siCRT on the cell cycle was examined by flow cytometry assay. As shown in Figure 3B, treatment with siCRT resulted in a decreased rate of cells in G2/M phase and an increased proportion of cells in G0/G1 phase in GBC-SD cell. However, there is no significant changes in cell cycle after treatment with siCRT in NOZ cells.

### Knockdown of CRT inhibits cell migration by inhibiting MMP-9 expression in GBC-SD and NOZ cells

**Figure 4:**
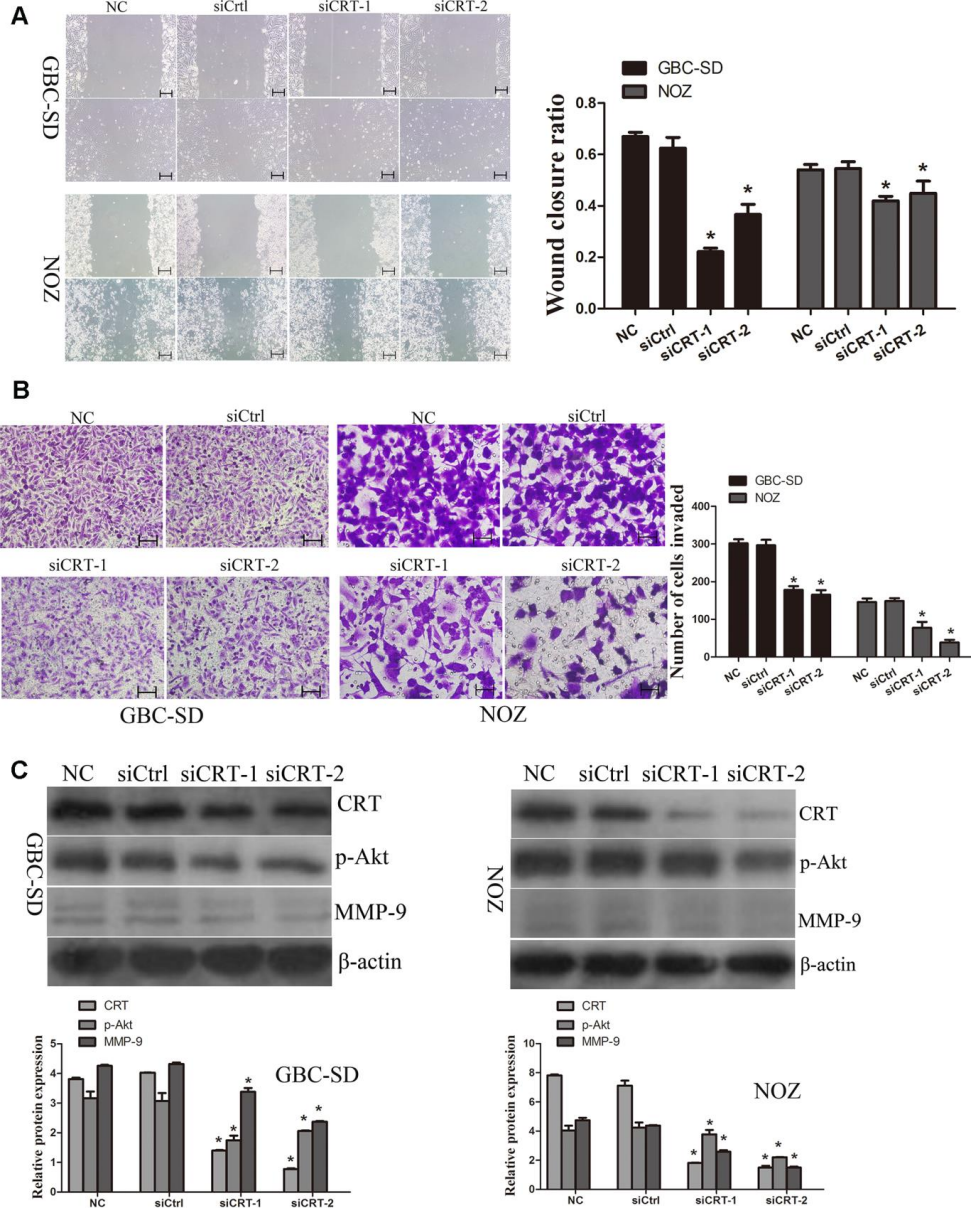
**Knockdown of CRT significantly inhibits cell migration *in vitro*.** (**A**) Cell migratory ability was detected by wound healing assay. (**B**) Cell migratory ability was detected by Transwell assay. Scale bars =200um. (**C**) Western blot assay revealed that knockdown of CRT inhibited the expression of p-Akt and MMP-9 in GBC-SD and NOZ cells.^*^P<0.05 vs. negative control. p-, phosphorylated; CRT, calreticulin; GBC, gallbladder cancer.

Since previous studies suggested that CRT may play a crucial role in the migration of cancer cells, the role of CRT in cell migration was examined in the present study. As shown in Figure 4A, the wound closure ratio in the NC and siCtrl groups in GBC-SD and NOZ cells after 48h were 0.67±0.02,0.58±0.02; 0.54±0.02, 0.54±0.03, whereas the wound closure ratio in the siCRT-1 and siCRT-2 groups after 48h were 0.22±0.01, 0.37±0.04; 0.41±0.02, 0.44±0.04, respectively. The present results suggested that the wound healing ratio was significantly decreased after transfection with siCRT. In addition, cell migration was also measured using a Transwell assay (Figure 4B). The number of migrated cells per field of view in the NC, siCtrl, siCRT-1 and siCRT-2 groups in GBC-SD and NOZ cells were 302±11, 297±15, 178±10 and 165±12; 146±10, 148±8, 77±15 and 37±7, respectively. The present data suggested that knockdown of CRT inhibited cell migration. Furthermore, the expression of p-Akt and MMP-9 was detected by western blot. As shown in Figure 4C, knockdown of CRT expression decreased the protein expression level of p-Akt and MMP-9.

### Overexpression of CRT significantly increases cell migration and decreases chemosensitivity of GBC-SD and NOZ cells to GEM through regulation of the PI3K/Akt pathway

**Figure 5:**
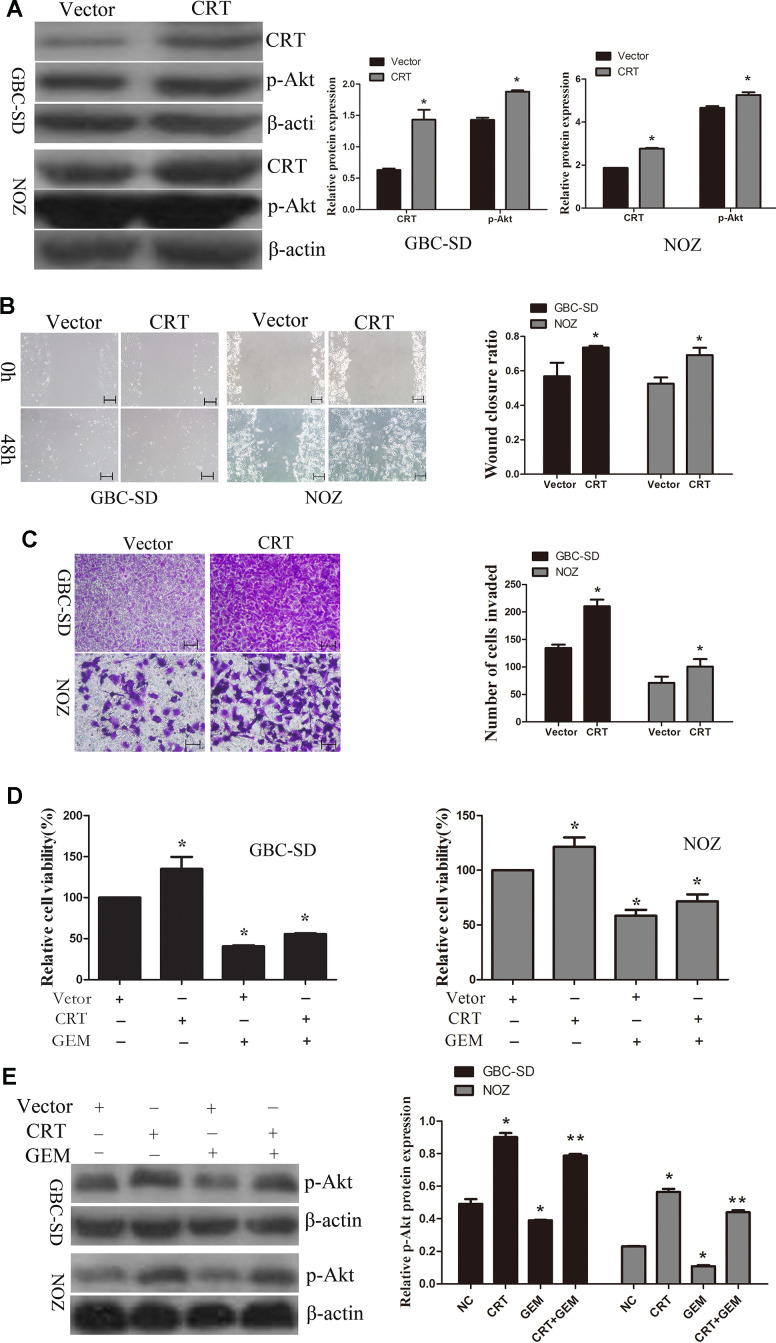
**Overexpression of CRT significantly increases cell migration and decreases chemosensitivity of GBC-SD and NOZ cells to GEM.** (**A**) CRT overexpression was confirmed by western blotting. (**B**) Cell migratory ability was detected by wound-healing assay. (**C**) Cell migratory ability was detected by Transwell assay. scale bars =200um. (**D**) Cell Counting Kit-8 assay was performed to analyze the proliferation of GBC-SD and NOZ cells. (**E**) Western blot assay suggested that p-Akt expression was involved in the chemosensitivity of GBC.^*^P<0.05 vs. negative control. p-, phosphorylated; CRT, calreticulin; GBC, gallbladder cancer; GEM, gemcitabine.

Next, CRT was transfected in GBC-SD and NOZ cells. As shown in Figure 5A, overexpression of CRT increased the levels of p-Akt in GBC-SD an NOZ cells. Therefore, additional experiments were performed to examine the role of CRT in GBC-SD and NOZ cells. As shown in Figure 5B, 5C, upregulation of CRT significantly increased cell migration, as assessed by wound healing and Transwell assays. GEM is currently the first-line chemotherapeutic drug for bile tract cancer and pancreatic cancer. Accumulating evidence demonstrated that GEM treatment for cancer patients remains unsatisfactory. As shown in Figure 5D, overexpression of CRT could partly reversed GEM-mediated anti-proliferative effect. To further investigate the underlying mechanism of CRT in enhancing the sensitivity of GBC to GEM, the activation of the PI3K/Akt pathway was investigated. As shown in Figure 5E, knockdown of CRT suppressed the activation of Akt in GBC-SD and NOZ cells and enhanced the sensitivity of GBC cells to GEM.

### GEM inhibits tumor growth *in vivo* and suppresses the expression of levels of CRT and p-Akt

**Figure 6:**
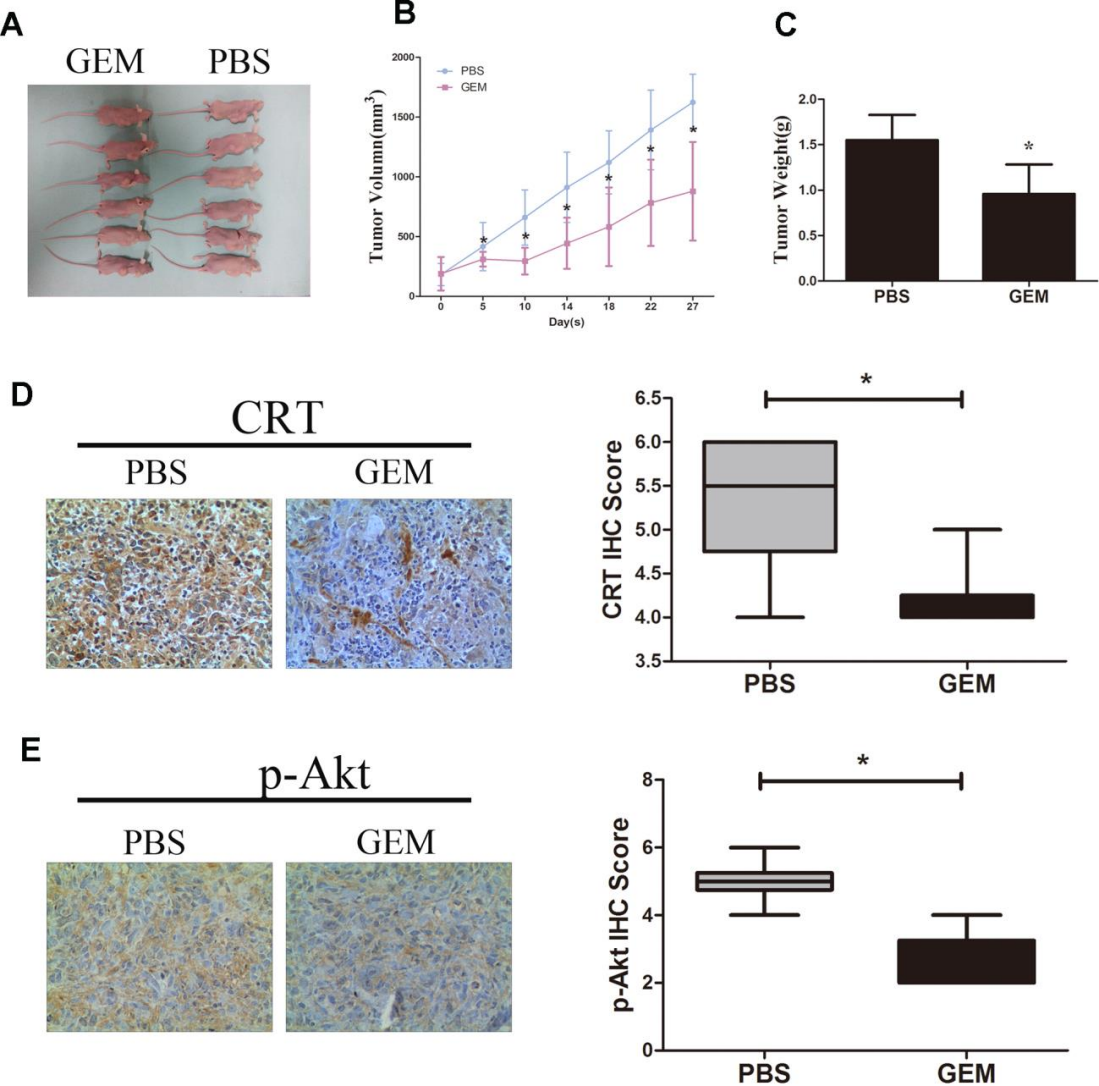
**GEM inhibits tumor growth and downregulates the expression of CRT and p-Akt in GBC-SD xenografts.** (**A**) Xenografted tumors derived by GBC-SD cells treated with PBS or GEM. (**B**) Growth curvesin mice are presented. (**C**) Tumor weights were measured at the endpoint of the animal experiments. (**D**, **E**) Expression levels of CRT and p-Akt in xenograft tumor were detected by IHC.^*^P<0.05 vs. negative control. P-, phosphorylated; CRT, calreticulin; GBC, gallbladder cancer; GEM, gemcitabine.

As shown in Figure 6A–6C, treatment with GEM significantly suppressed the growth and weight of tumors xenografted in immunodeficient BALB/c nude mice following subcutaneous injection of GBC-SD cells. In addition, treatment with GEM led to a decrease in the protein expression levels of CRT and p-Akt as assessed by IHC (Figure 6D, 6E), which was consistent with the aforementioned *in vitro* results.

## DISCUSSION

Previous studies showed that CRT is upregulated in pancreatic cancer [15], hepatocellular carcinoma [16], lung cancer [23] and is involved in tumor prognosis. However, little is known regarding its expression and mechanism in GBC. In the present study, the expression level of CRT was found to be upregulated in GBC and correlated with tumor size. However, its expression was not correlated with additional clinicopathological parameters, such as lymphatic nodes and TNM state.

To investigate the possible biological functions of CRT in GBC, the expression of CRT was examined in 32 GBC tissues by RT-qPCR and IHC assays. The present data suggested that CRT was markedly upregulated in 25 of 32 cases of GBC tissues compared with their adjacent non-tumor tissues and chronic cholecystitis tissues. In addition, there was a significant positive correlation between CRT expression level and aggressiveness of GBC.

Previous studies have shown that CRT was involved in mediating tumor growth and migration, and drug resistance. In addition, previous studies showed that CRT regulates the development and progression of myeloproliferative neoplasms [18, 19], suggesting that CRT may serve an important role in tumor progression. In line with previous studies, the present data showed that CRT acts as an oncogene in GBC. To further examine the biological functions in GBC, the effects of CRT on cell proliferation and colony formation were investigated in GBC-SD and NOZ cells by CCK-8 and colony formation assays, respectively. Knockdown of CRT inhibited cell proliferation and colony-forming ability. In addition, the present data suggested that knockdown of CRT inhibited tumor growth in an *in vivo* model. To investigate the underlying mechanisms of growth inhibition, apoptosis was investigated in GBC-SD and NOZ cells by flow cytometry after knockdown of CRT. As expected, inhibition of CRT induced cell apoptosis. The effects of CRT knockdown on GBC-SD and NOZ cell cycle progression were also evaluated. Knockdown of CRT induced cell cycle arrest in G0/G1 phase in GBC-SD cells. Herein, when we analyzed data from cell cycle in NOZ cells, apoptotic cells were not included, so there was no significant changes in cell cycle progression in NOZ cells. In addition, the effects of CRT on cell migration were examined in GBC-SD and NOZ cells by wound healing and Transwell assays. Inhibition of CRT significantly decreased the migratory ability of GBC-SD and NOZ cells, whereas overexpression of CRT promoted cell migration in GBC-SD and NOZ cells. Cancer cell migration is a complex process that requires several physiological changes [24]. MMPs are members of the zinc-dependent endopeptidases [25]. Accumulating evidence suggested that MMPs are upregulated in many types of cancer, including GBC [26], and play a crucial role in tumor progression. In the present study, inhibition of CRT decreased the expression and secretion of MMP-9, suggesting that MMP-9 may be involved in the CRT-mediated cell migration.

Recently, a number of studies have shown the contribution of CRT to chemoresistance in various types of human cancer [15, 27]. In line with previous studies, the present data showed that inhibition of CRT increased the chemosensitivity of GBC-SD and NOZ cells to GEM. However, further studies are needed to examine the mechanisms underlying GEM. The PI3K/Akt pathway serves an important role in chemoresistance and its activation has been shown to increase the chemoresistance in cancer cells [28, 29]. In the present study, inhibition of CRT decreased the activation of p-Akt, whereas overexpression of CRT promoted the activation of p-Akt and decreased the chemosensitivity of GBC-SD and NOZ cells to GEM. Taken together, the present results suggested that CRT may represent a potential diagnostic and therapeutic biomarker in GBC. Previous study showed that somatic mutations in CRT gene were identified in Janus kinase 2 gene (JAK2 V617F)-negative or MPL-negative primary myelofibrosis (PMF) and essential thrombocythemia (ET) patients. CRT trails JAK2 as the second most mutated gene in myeloproliferative neoplasms (MPNs). However, little is known about CRT mutation in solid tumor, including gallbladder cancer.

In conclusion, the present results suggested for the first time that CRT is frequently overexpressed in GBC tissues, and its expression may be associated with tumor size in patients with GBC. Inhibition of CRT suppressed cell proliferation and migration, induced apoptosis and blocked cell cycle progression. In addition, inhibition of CRT repressed the activation of p-Akt, which in turn enhanced the anti-cancer efficacy of GEM.

## MATERIALS AND METHODS

### Tumor and adjacent tissue samples

Tumor and adjacent non-tumor tissues were collected from 32 patients with GBC after cholecystectomy in the First Affiliated Hospital of Zhengzhou University between June 2016 and April 2017. Normal gallbladder tissues from patients with chronic cholecystits were used as control. The present study was approved by the Clinical Research Ethics Committee of the First Affiliated Hospital of Zhengzhou University. Complete clinicopathological and laboratory data were collected from all subjects enrolled in the present study. Tissues were immediately snap-frozen in liquid nitrogen after surgical resection and stored at -80° C prior to further analysis. Patients who received radiotherapy, chemotherapy or immunotherapy before surgery were excluded from the present study.

### Cell culture

Human GBC cells GBC-SD were purchased from the Shanghai Cell Bank (China Academy of Science) and NOZ were obtained from Procell Life Science and Technology Co., Ltd (Wuhan, China). GBC-SD were cultured in RPMI-1640 (Solarbio Life Sciences) medium and NOZ were cultured in DMEM/F12(HyClone; GE Healthcare Life Sciences) medium supplemented with 10% FBS (GemCell), 100 U/ml penicillin and 100 mg/l streptomycin under a humidified atmosphere of 5% CO_2_ at 37° C.

### Cell counting Kit-8 (CCK-8) cell proliferation and colony formation assay

Cell proliferation was detected by CCK-8 (Dojindo Molecular Technologies, Inc.). GBC-SD and NOZ cells were seeded in 96-well plates at a density of 3x10^3^ cells/well. After treatment, the cells were incubated with 10 μl CCK-8 solution for 1 h. The optical density values were measured by assessing the absorbance at a wavelength of 450 nm using a micro-plate reader. For the colony formation assay, 1x10^3^ cells were seeded into six-well plates and cultured for 14 days. Subsequently, the colonies were stained with 0.1% crystal violet. The number of colonies with >50 cells was counted as previously described.

### Cell apoptosis and cell cycle analyses by flow cytometry

For the cell apoptosis analysis, GBC-SD and NOZ cells were collected after the indicated treatment. Cells were washed twice with cold PBS and resuspended at a density of 1x10^6^cells/ml. Subsequently, 100 μl of binding buffer was incubated with 5 μl Annexin V-FITC and 5 μl propidium iodide (BD Biosciences) in the dark at room temperature for 15 min. For the cell cycle analysis, cells were collected and fixed in 70% cold ethanol overnight at 4° C. Then, cells were stained with propidium iodide at 37° C for 30 min. Cell apoptosis and cell cycle were detected with an Epics XL-MCL ADC flow cytometer (Beckman Coulter, Inc.) or FACS Calibur system (BD Biosciences, San Jose, CA, USA) according to the manufacturer’s instructions. Data were analyzed using an EXP032ADC system or BD FACS Diva 8.0.1 software.

### Wound healing assays

GBC-SD and NOZ cells were seeded into six-well plates and cultured overnight. After cells were seeded, a vertical wound was made, and cells were washed with PBS twice. Images were obtained before incubating the cells at 37° C. Then, GBC-SD or NOZ cells were cultured with 2% FBS and imaged after 48 h. Wound closure ratio was calculated as follows: (width at 0 h - width at 48 h)/width at 0 h.

### Cell migration

GBC-SD and NOZ cells were suspended in 100 μl serum-free medium and added to the upper chamber. In addition, 600 μl 10% FBS medium were added to the lower Transwell chamber (Corning, Inc.). After incubation for 24 h, cells attached to the lower surface of the membrane were washed with PBS, fixed in 100% methanol for 20 min and stained with 0.1% crystal violet for 10 min. The cells migrated through the membrane were visualized with an inverted microscope (Olympus Corporation) and counted in five randomly selected fields of view.

### Cell transfection

CRT overexpression and CRT downregulation were performed by transfection with CRT lentivirus or small interfering (si)RNA (GenePharma Biotechnology,). Transfection was performed using Lipofectamine 2000 reagent (Invitrogen; Thermo Fisher Scientific Inc.) following the manufacturer’s protocol. The sequences of the oligonucleotides used were as follows: siCRT-1 sense, 5’-GUGACGAGGAGAAAGAUAATT-3’ and anti-sense, 5’-UUAUCUUUCUCCUCGUCACTT-3’; siCRT-2 sense, 5’-GCAAGAACGUGCUGAUCAATT-3’ and anti-sense, 5’-UUGAUCAGCACGUUCUUGCTT-3’; si-control (Ctrl) sense, 5’-UUCUCCGAACGUGUCACGUTT-3’ and anti-sense, 5’- ACGUGACACGUUCGGAGAATT-3’.

### Immunohistochemistry (IHC)

Slides were immersed in heated antigen retrieval solution (10 mmol/l citrate buffer; pH 6.0), cells were then treated with 3% H_2_O_2_ for 10 min. After washing with PBS, the slides were incubated with diluted primary antibodies anti-CRT (1:100) and anti-phosphorylated (p-)Akt (1:100) at 4° C overnight. Then, the membranes were incubated with secondary antibodies (1:500; ZSGB-BIO) for 20 min at room temperature. The reaction was developed using a 3,3’-diaminobenzidine kit (1:50 dilution). Finally, the slides were counterstained in hematoxylin before dehydration and mounting. Staining was observed by microscopic examination.

### RNA isolation and reverse transcription-quantitative(RT-q)PCR

Total RNA was extracted from tissue samples using TRIzol (Invitrogen; Thermo Fisher Scientific, Inc.) according the manufacturer’s protocol. RNA was retrotranscribed to cDNA according to the manufacturer’s protocol using the Takara PrimeScript First Strand cDNA Synthesis kit (Takara Bio, Inc.). RT-qPCR analysis was performed using an Agilent MX 3005P system with a SYBR Green PCR Amplification kit (Takara Bio, Inc.). Each qPCR analysis was performed in triplicate. β-actin served as control. The primers used were as follows: CRT sense,5’-GGAGCAGTTTCTGGACGGAG-3’ and anti-sense 5’-ACCGTAGAACTTGCCGGAAC-3’; andβ-actin sense5’-CCTGGCACCCAGCACAAT-3’ and anti-sense 5’-GGGCCGGACTCGTCATAC-3’.

### Western blot analysis

Total proteins from cells were extracted with ice-cold RIPA buffer for 20 min. Protein concentration was determined by a bicinchoninic assay kit (Beyotime Institute of Biotechnology). In total, 30 μg of protein from each sample was subjected to 10% SDS-PAGE, and transferred onto PVDF membranes (EMD Millipore). The membranes were blocked in PBS containing 5% non-fat dry milk for 1 h at room temperature and hybridized with the primary antibodies anti-CRT (1:300) anti-matrix metallopeptidase 9 (MMP-9; 1:1,000) anti-p-Akt (1:1,000) and anti-β-actin (1:5,000). All primary antibodies were purchased from Proteintech Group. Primary antibodies were incubated overnight at 4° C, and membrane was washed four times for 10 min with PBS. Then, the membranes were incubated with horseradish peroxidase-conjugated goat anti-rabbit IgG (1:5,000) for 1 h at room temperature and washed four times with PBS. Protein bands were detected using an enhanced chemiluminescence western blotting detection kit (Thermo Fisher Scientific, Inc.) according to the manufacturer's protocol.

### Xenograft model experimental

GBC-SD cells in log-phase growth were resuspended in serum-free culture medium at a density of 5x10^6^ cells in 200 μl. Then, tumor xenografts were established by subcutaneous injection of these GBC-SD cells into the right flank of nude mice. The mice were randomly divided into four groups (n=5 in each group). The volume of the tumors was measured every 4 days. Tumor volume was calculated using calipers and estimated according to the following formula: Tumor volume (mm^3^) = (ax b^2^)/2, where ‘a’ and ‘b’were the longest and shortest diameters, respectively. After 24 days, the animals were sacrificed, and the tumor tissue was removed and measured. Xenograft tumors were harvested and cut into sections for IHC analysis. The experimental procedure involving animal tests was conducted in accordance with the National Institutes of Health Guide for Care and Use of Laboratory Animals and local institutional ethical guidelines. The animal protocols were approved by the Institutional Animal Care and Use Committee of The First Affiliated Hospital of Zhengzhou University.

### Statistical analysis

Results are presented as the mean ± SD from three independent experiments. Analysis was performed using SPSS 17.0 software (SPSS Inc.). Differences between groups were assessed using Student’s t-test, ANOVA or χ^2^ test. Kaplan-Meier method was used to establish survival cures for OS. The differences in survival of patients between high and low CRT expression were assessed by Log-rank test. The univariate and multivariate Cox’s proportional hazards regression analyses were used to determine prognostic factors of OS. P<0.05 was considered to indicate a statistical significant difference.
